# *Lactobacilli* and *Bifidobacterium* as anti-atherosclerotic agents 

**DOI:** 10.22038/IJBMS.2022.63860.14073

**Published:** 2022-08

**Authors:** Milad Abdi, Hadi Esmaeili Gouvarchin Ghaleh, Reza Ranjbar

**Affiliations:** 1 Research Center of Tropical and Infectious Diseases, Kerman University of Medical Sciences, Kerman, Iran; 2 Applied Virology Research Center, Baqiyatallah University of Medical Sciences, Tehran, Iran; 3 Molecular Biology Research Center, Systems Biology and Poisonings Institute, Baqiyatallah University of Medical Sciences, Tehran, Iran

**Keywords:** Atherosclerosis, Bifidobacterium Hypercholesterolemia, Inflammation, Lactobacilli, Oxidative stress, TMAO

## Abstract

Atherosclerosis is the thickening or hardening of the arteries which is caused by a buildup of atheromatous plaque in the inner lining of an artery. Hypercholesterolemia, inflammation, oxidative stress, and trimethylamine N-oxide (TMAO) are important risk factors for atherosclerosis. Therefore, this study aimed to review the anti-atherosclerotic effects of Lactobacilli and *Bifidobacterium* via improving lipid profile and reducing the effects of oxidative stress, inflammation, and TMAO. To prepare the present review, several databases such as Scopus, PubMed, and Google Scholar were searched, and relevant articles from 1990 until 2022 were selected and reviewed. The present review showed that *Lactobacilli* and *Bifidobacterium* reduce the risk of atherosclerosis in both *in vitro* and *in vivo* studies by breaking down or altering cholesterol metabolism with the help of their by-products and by reducing inflammation and oxidative stress and TMAO. Consumption of Lactobacilli and Bifidobacterium can be useful in prevention of atherosclerosis.

## Introduction

Atherosclerosis is the thickening or hardening of the arteries that is caused by a buildup of atheromatous plaque in the inner lining of an artery. Atheromatous plaque is made up of deposits of fatty substances, cholesterol, cellular waste products, calcium, and fibrin (1). Atherosclerosis is a slow and progressive process that usually develops over several years through a complex series of cellular events occurring within the arterial wall and in response to a variety of local vascular circulating factors (2). Cardiovascular diseases (CVDs), which are predominantly the clinical manifestation of atherosclerosis, are the leading cause of death in all regions of the world except Africa (3). CVDs led to 17.3 million deaths in 2008 and it is estimated that by 2030, more than 23.3 million deaths will occur annually in the world (4). Coronary heart diseases (CHDs) are the most common cardiovascular diseases (CVDs) (5). CHDs burden costs $108.9, $23, £30, $94, and €196 billion per year in the United States, Canada, UK, Australia, and Europe, respectively (6-8). Hypercholesterolemia, inflammation and oxidative stress, and trimethylamine N-oxide (TMAO) are important risk factors for atherosclerosis (1, 2, 9). 

Hypercholesterolemia is elevated levels of non-high-density lipoprotein cholesterol (HDL-C) (subtraction of HDL-C from total cholesterol (TC)), particularly low-density lipoprotein cholesterol (LDL-C) (10). The low intake of high-fat diets, free sugar, and carbohydrates can reverse hypercholesterolemia and the risk of CVDs. The treatment of hypercholesterolemia is expensive with severe drawbacks (11). In the USA, about one-third of the hypercholesterolemic subjects are under control and only half of them can be treated (11). Studies have demonstrated that the risk of CHDs and CHDs-related mortality increase by about 35% and 45%, respectively for every 1 mmol increase in serum cholesterol levels; and the risk of CHDs decrease by 2%–3% for every 1% decrease in serum cholesterol levels (12). 

The microbial flora of the gastrointestinal (GI) tract of humans and animals plays important roles in various functions. Recently, many scientists have been interested in the use of this useful microbial flora for sustaining and promoting human health (13). 

Based on World Health Organization (WHO) definition, probiotics are “live microorganisms when administered in adequate amounts confer a health benefit on the host”. These useful microorganisms are used in different forms such as yogurt, cheese, and fermented foods as well as capsules, powders, etc., as foods or therapeutic agents for health purposes (4, 13). 

Elie Metchnikoff is the first person that declared the useful benefits of probiotics. He believed that lactic acid bacteria (LAB), an important group of probiotics, contained in sour milk are the cause of the longevity of Bulgarian peasants and the inhibition of pathogens (13). 

*Lactobacilli *and *Bifidobacterium *are the most well-known bacterial genera of probiotics which are generally regarded as safe (GRAS) and called friendly bacteria (14-16). It has been shown that several strains of *Lactobacilli* and *Bifidobacterium* are available for human use which have several health benefits, including improvement of intestinal health, amelioration of lactose intolerance symptoms, reduction of the risk of various other diseases such as infectious diseases and cancer (17, 18). These microorganisms are able to reduce the risk of CVDs)by reducing the serum level of total cholesterol and LDL as well as by increasing the serum level of HDL (19, 20). There are studies that have shown the beneficial effects of these microorganisms on atherogenic factors like inflammation and oxidative stress and TMAO (21, 22). Therefore, this study aimed to review the anti-atherosclerotic effects of *Lactobacilli* and *Bifidobacterium* via improving the lipid profile and reducing the effects of oxidative stress, inflammation, and TMAO in *in vitro* and *in vivo* (animal and human) studies, with focus on cholesterol-lowering properties.

***Effects on cholesterol***

*Cholesterol metabolism*

Cholesterol is an organic molecule that is used in the membrane structure of eukaryotic cells and serves as the precursor of bile acids (BAs), corticosteroids, steroid hormones, and vitamin D. About 75% of the pooled body cholesterol is synthesized by the mevalonate pathway and the remaining 25% is obtained from foods (23, 24). Many enzymes and transporters are involved in cholesterol metabolism in the intestine and liver. Niemann-Pick C1-like 1 (NPC1L1) is an important transporter located on enterocytes and hepatocytes which transport dietary cholesterol into the intestine and cholesterol from bile, respectively (25, 26). LDL receptors (LDLR) are located on polarized cells’ surfaces and take up circulating blood cholesterol. The endogenous synthesis of cholesterol is performed in the liver by the mevalonate pathway and starts with the two molecules of acetyl coenzyme A (acetyl-CoA). More than 20 enzymes catalyze this pathway, of them, 3-hydroxy-3-methylglutaryl coenzyme A reductase (HMGCR) is the rate-limiting enzyme of this pathway (27). There is an important transcription factor, sterol-regulatory element-binding protein 2 (SREBP-2) regulating the uptake and biosynthesis of cholesterol (28).

After cholesterol formation, it is converted to cholesterol ester (CE) and stored in lipid droplets or used in lipoproteins. ATP-binding cassette sub-family G member 5 and member 8 (ABCG5/8) transport excess cholesterol to the intestine or the bile (27). Due to the hydrophobicity of cholesterol, it is packaged within lipoproteins to be transported effectively throughout the body. There are four major types of lipoproteins in blood including chylomicrons, very-low-density lipoproteins (VLDL), LDL, and HDL(27).

Chylomicrons are the least dense cholesterol transport molecules that contain apolipoprotein B-48, C, and E and transport exogenous cholesterol from the intestine to muscles and other tissues. After intestinal absorption of dietary cholesterol by NPC1L1, it is esterified to CE and then packaged with triglycerides and apolipoproteins into chylomicrons (29). Under the action of lipoprotein lipase, chylomicrons are converted to chylomicron remnants which are removed by the liver but a small amount remains and can penetrate the endothelial monolayer of an artery and participate in plaque buildup which increases the risk for atherosclerosis (30).

VLDL particles are synthesized by the liver and contain apolipoprotein B100 and E, triglycerides, and cholesterol. This type of lipoprotein can be degraded by lipoprotein lipase and converted to intermediate-density lipoproteins (IDL) and then metabolized to cholesterol-enriched LDL. LDL particles are the major carriers of cholesterol in the blood which through LDL receptors transport cholesterol to a variety of tissues such as the wall of the artery which can promote atherosclerosis. In addition, LDL particles can directly bind to endothelial scavenger receptor class B type 1 (SR-B1) and penetrate to the subendothelial space of arteries then be oxidized, finally causing a buildup of immune cells and atherosclerotic plaque formation (31, 32). Macrophages internalize oxidized LDL particles to form foam cells that often are trapped in the walls of arteries and contribute to forming atherosclerotic plaque. The atherosclerotic plaques are the main cause of CVD (32, 33). HDL particles transport cholesterol from tissues to the liver, either for excretion or for other uses, in a process known as reverse cholesterol transport (RCT) (30). 

The excess cholesterol in macrophages can also be excreted by the ATP-binding cassette subfamily A member 1 (ABCA1) or subfamily G member 1 (ABCG1) which prevents the formation of foam cells thereby reducing the risk of atherosclerosis and CVD (34). 

**Figure 1:**
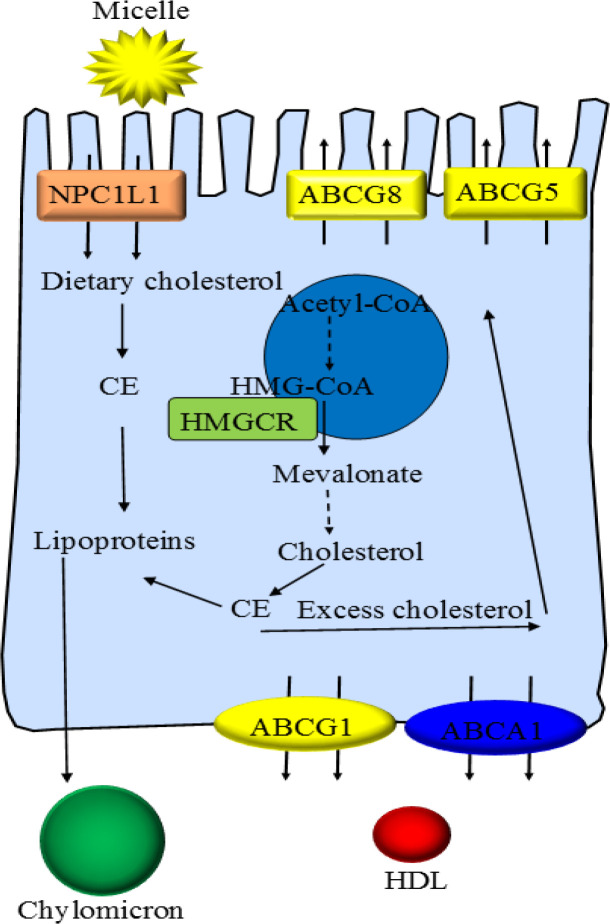
The schematic of cholesterol metabolism. NPC1L1, Niemann-Pick C1-like 1; CE, cholesterol ester; ABCG5, ATP-binding cassette sub-family G member 5; ABCG8, ATP-binding cassette sub-family G member 8; ABCG1, ATP-binding cassette sub-family G member 1; ABCA1, ATP-binding cassette subfamily A member 1; HMG-CoA, 3-hydroxy-3-methylglutaryl coenzyme A; HMGCR, 3-hydroxy-3-methylglutaryl coenzyme A reductase

When intracellular cholesterol is increased, liver X receptors (LXR) –ligand-activated transcription factors of the nuclear receptor superfamily– are activated and mediate cholesterol efflux through increased expression of the ABCG5/8 transporters (35, 36). Figure 1 shows the metabolism of cholesterol and its import and export in a polarized cell.

***Bile acids***

**Figure 2:**
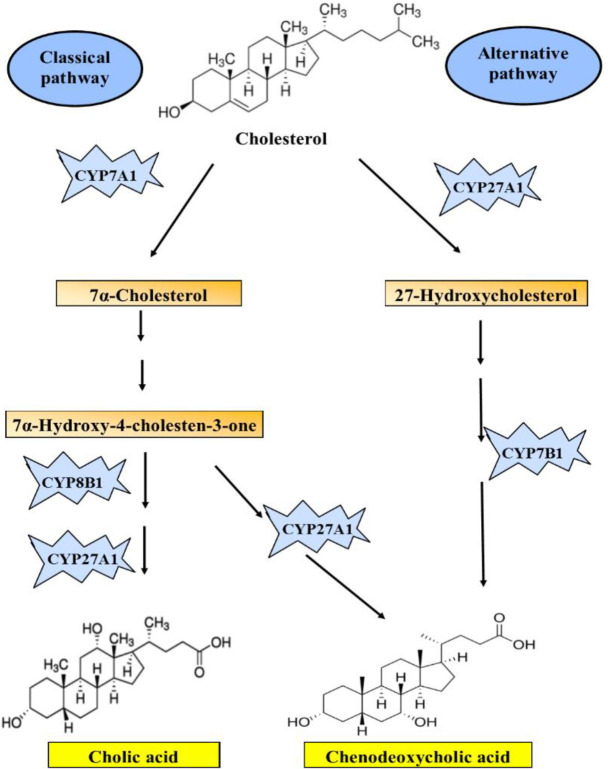
The schematic of bile acids synthesis (cholic acid and chenodeoxycholic acid) from cholesterol.CYP7A1, cholesterol 7α-hydroxylase; CYP27A1, sterol 27-hydrolyase; CYP7B1, oxysterol 7α-hydroxylase; CYP8B1, sterol 12α-hydroxylase

Bile acids (BAs) are synthesized from cholesterol via the classical pathway or alternative pathway (Figure 2). The classical pathway is the main production pathway of BAs (90%) in which cholesterol is converted to BAs by the rate-limiting enzyme cholesterol 7 α-hydroxylase (CYP7A1) in the liver. In the alternative pathway, the remaining 10% BAs are produced by sterol 27-hydrolase (CYP27A1) in extrahepatic sites including the vascular endothelium and macrophages (37). There are well-known feedback loops that control gene transcription of the key enzymes in BA synthesis. The nuclear farnesoid X receptor (FXR) is one of them that in an increased BA pool induces the expression of small heterodimer partner (SHP), thereby down-regulates CYP7A1expression (38). First, primary BAs, cholic acid, and chenodeoxycholic acid are synthesized and then conjugated with glycine or taurine residues to intestinal bile salts. Conjugated BAs are transported into bile by the bile salt export pump (BSEP) or by ABCG5/8 (39). These conjugated BAs are stored temporarily in the gallbladder until they are released into the small intestine upon meals to facilitate emulsion.

About 95% of bile acids are reabsorbed by active transport mainly via the apical sodium-dependent bile acid transporter (ASBT) from the ileum and secreted into the portal circulation by the organic solute transporters (OST α/β) and recycled back to the liver for further secretion into the biliary system and gallbladder. Increased levels of BAs in the ileum can activate FXR inhibiting ASBT and reabsorption of BAs. In addition, activation of FXR induces the production and secretion of fibroblast growth factor 19 (FGF19) which binds to fibroblast growth factor receptor 4 (FGFR4) and activates a signaling pathway that down-regulates CYP7A1 (40). 

***Mechanisms for cholesterol reduction***

**Figure 3:**
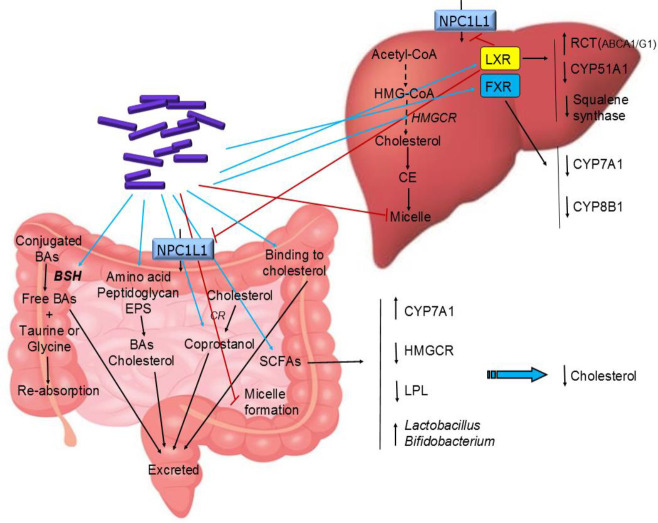
Cholesterol-lowering mechanisms of Lactobacilli and Bifidobacterium. Blue and red arrows show increase and decrease in activity/expression, respectively. CYP7A1, cholesterol 7α-hydroxylase; CYP8B1, sterol 12α-hydroxylase; CYP51A1, lanosterol 14α-demethylase; RCT, reverse cholesterol transport; FXR, farnesoid X receptor; LXR, Liver X receptor; EPS, exo-polysacharides; CR, cholesterol reductase; BAs, bile acids; SCFAs, short-chain fatty acids; BSH, bile salt hydrolase; LPL, lipoprotein lipase; NPC1L1, Niemann-Pick C1-like 1; CE, cholesterol ester; ABCG1, ATP-binding cassette sub-family G member 1; ABCA1, ATP-binding cassette subfamily A member 1; HMG-CoA, 3-hydroxy-3-methylglutaryl coenzyme A; HMGCR, 3-hydroxy-3-methylglutaryl coenzyme A reductase

There are several possible mechanisms for the cholesterol-lowering effect of *Bifidobacterium* and *Lactobacilli* which are obtained based on *in vitro* and animal studies (Figure 3). These mechanisms include deconjugation of BAs by bile salt hydrolase (BSH), binding to cholesterol and incorporation into the cellular membrane (41, 42), production of short-chain fatty acids (SCFAs) (33, 43), trapping cholesterol by peptidoglycan and exopolysaccharides (44, 45), and conversion of cholesterol to coprostanol (46). 

The most profound cholesterol-lowering mechanism has been attributed to bile salt hydrolase (BSH) activity (41). Studies have shown that many *Bifidobacterium *and* Lactobacilli *species produce BSH which can cleave amide bonds of conjugated BAs and convert them to liberate free primary BAs and amino acid (taurine or glycine) in which BAs are more hydrophobic and less efficiently reabsorbed from intestines and thereby excreted in feces (41, 47-49). Therefore, BSH activity causes an increase in the production of BAs from cholesterol, thus leading to the reduction of cholesterol. Moreover, following the activity of BSH, due to low BAs, the formation of micelles is disrupted, thereby the cholesterol absorption in the intestine is decreased (50).

Another cholesterol-lowering mechanism of *Lactobacilli* and *Bifidobacterium* is the ability to bind cholesterol and incorporate it into cell membranes (51) The ability of cholesterol-binding appeared to be growth and strain-specific. It has been observed that, although live bacterial cells lower more cholesterol than dead cells, the dead bacterial cells can remove cholesterol from media, which indicates cholesterol can also be bound to the cellular surface (52). Some *Lactobacilli* species have been found to possess surface protease-sensitive receptors which bind to exogenous cholesterol or phosphatidylcholine vesicles and incorporate them into their cell membranes (42, 53).

The cell wall amino acids and peptidoglycan as well as the Exo-polysaccharides (EPS) of probiotics have a role in the reduction of cholesterol (44, 45). Cholesterol-lowering by this mechanism is however strain and growth-dependent (52). Some intestinal microorganisms such as *Lactobacilli* and *Bifidobacterium* species can synthesize different EPS (54-56). It has been observed that the amount of produced EPS is correlated with the quantity of assimilated cholesterol by probiotic strains. The EPS of *Lactiplantibacillus plantarum* BR2 and *Lacticaseibacillus paracasei* M7 showed a cholesterol-lowering effect (57). 

Short-chain fatty acids (SCFAs) are important components produced from oligosaccharides by gut microbiota and also probiotic strains such as *Lactobacilli* and *Bifidobacterium* species which have been found to play a vital role in cholesterol reduction (43, 58, 59). It has been reported that SCFAs are able to activate peroxisome proliferator-activated receptors (PPARs). The peroxisome proliferator-activated receptor γ (PPARγ) controls angiopoietin-like protein 4 (ANGPTL4) which inhibits lipoprotein lipase (LPL) (60). Therefore, SCFAs suppress LPL activities by activation of PPARγ and up-regulating ANGPTL4 levels which leads to regulation of fatty acid oxidation in muscle and adipocytes that reduce fat storage (61, 62). It has been reported that butyrate is able to inhibit the synthesis of cholesterol by reducing HMG-CoA reductase (HMGCR) activity which is the rate-limiting enzyme of cholesterol synthesis (63). SCFA can also stimulate the hepatic synthesis of BAs by up-regulation of CYP7A1 which leads to reduction of cholesterol (64). A high concentration of SCFA in the gut can lower colonic pH and promote the growth of *Lactobacilli *and *Bifidobacterium *species (54, 65, 66).

Another mechanism is the conversion of cholesterol into coprostanol which reduces cholesterol absorption and makes it easily eliminable with defection (67). The efficiency of this mechanism depends on the abundance of bacteria that possess reductase enzymes. This enzyme is present in some species of *Lactobacilli* and *Bifidobacterium* species such as *L. acidophilus* ATCC 314, *L. acidophilus* FTCC 0291, *L. bulgaricus* FTCC 0411, and *Bifidobacterium bifidum* PRL2010 (64). In another study, it has been observed that *L. acidophilus, L. bulgaricus and Lacticaseibacillus casei *ATCC 393 contain both intracellular and extracellular cholesterol reductase (20, 42). 

Liver X receptors (LXRs) can be stimulated by *Lactobacilli *and *Bifidobacterium *species which down-regulate NPC1L1 expression in the liver and intestine thereby reducing cholesterol absorption in the intestines and re-absorption in the liver (68-70). LXRs also down-regulate two genes, squalene synthase and lanosterol 14α-demethylase (CYP51A1) genes that encode key enzymes in the synthesis of cholesterol (71). In a study, *L. plantarum *lowered cholesterol levels via up-regulation of LXR (72, 73). LXR also plays an important role in RCT by regulating several transporters including ABCA1 and ABCG1, so can help to reduce cholesterol and atherosclerosis (74). NPC1L1 acts as a negative regulator of NPC2, therefore down-regulation of NPC1L1 up-regulates NPC2 which in return increases cholesterol exporters such as ABCG5/8 (75). 

Also, the farnesoid X receptor (FXR), a bile acid nuclear receptor may be stimulated by deconjugated BAs. FXR senses the increased level of hepatic BAs and induces the expression of SHP which down-regulates rate-limiting enzymes CYP7A1 and sterol-12α-hydroxylase (CYP8B1) (76, 77). 

***In vitro and animal studies*** ***on cholesterol-lowering***

Numerous studies have been performed on the cholesterol-lowering effect of *Lactobacilli* and *Bifidobacterium* species most of which are *in vitro* and animal experiments. These experiments have shown significant efficacy of *Lactobacilli* and *Bifidobacterium* species on cholesterol reduction (20, 78, 79).

***Lactobacilli species***

*Lactobacilli* species have been shown in various studies to have a high ability to decrease cholesterol (78, 80-84) some of which are listed below.

*L. plantarum *is the most common *Lactobacilli* investigated in cholesterol-lowering efficacy. In a study, the effect of *L. plantarum* PH04 at a dose of 10^7^ colony-forming unit (CFU)/mouse daily for 14 days on serum cholesterol and TGs levels of hypercholesterolemic mice was investigated. *L. plantarum* PH04 resulted in 7% and 10% reduction in the serum cholesterol and TGs, respectively (82). Also, *L. plantarum* CK 102 at a dose of 5×10^7^ CFU/ml has significantly reduced the serum levels of total cholesterol and LDL-C in Sprague-Dawley rats (85). In some studies, the cholesterol-lowering ability of *L. plantarum* in three conditions of growing, resting, and death has been examined. For example, in an *in vitro* study, four strains of *L. plantarum* (TGCM 15, TGCM 26, TGCM 33, and TGCM 128) showed more than 50%, (13.11–23.28%), and (11.44–19.53%) cholesterol reduction in growing, resting, and dead cells, respectively (83). In a similar study, *L. plantarum* strains isolated from mustard removed cholesterol by growing (110.67 to 167.03 μg/ml), resting (1.40 to 35.44 μg/ml) and dead (0-54.21 μg/ml) cells (86). It has been shown that double-coated *L. plantarum* KCTC3928 with proteins and polysaccharides can significantly reduce the serum LDL-C and TGs levels in mice (87). Cholesterol-lowering ability of *L. plantarum *strains obtained from various sources such as kefir grains (47), traditional fermented milk (88), and local fermented food products (89) has been displayed. BSH activity and cholesterol coprecipitation are the most common mechanisms seen in *L. plantarum *strains (83, 85). Expression of the LDL-R and HMGCR were marginally affected and CYP7A1 was significantly up-regulated following live *L. plantarum* KCTC3928 feeding (87, 89).

Also, *L.* *acidophilus *has a remarkable cholesterol-lowering ability. In a study by Sarkar in 2003, the cholesterol-lowering ability of *L. acidophilus *was demonstrated. This study reported that cholesterol reduction is due to the assimilation or/and attachment of cholesterol to the surface of *L. acidophilus *cells (90). *L.* *acidophilus *also has shown BSH activity and co-precipitation with cholesterol ability, the ability that increases with decreasing pH (91). The cell-free supernatant (CFS) of* L.* *acidophilus *ATCC 43121 can reduce cholesterol in the broth in the presence of bile salts (92). 

*L. casei* is another *Lactobacilli* with cholesterol-lowering ability. *L. casei* has demonstrated that it is able to reduce serum cholesterol levels alone (93) or/and in combination with prebiotics (94).

There are other *Lactobacilli* species that have been less examined for their cholesterol-lowering activities. *Lactobacillus crispatus *isolated from gherkins (fermented cucumber) has significantly reduced cholesterol (95). *Lactobacillus bulgaricus* (96), *Limosilactobacillus reuteri* (97),* and Lactobacillus gasseri *SBT0270 (98) have significantly improved the lipid profile of rats, mice, and rats, respectively. In a study, *L. plantarum* 9-41-A and *Limosilactobacillus fermentum* M1-16 resulted in significant reduction in the serum levels of total cholesterol, LDL-C, and TGs in rats, whereas HDL-C level was not significantly changed (*P*>0.05) (99). In a similar study, the cholesterol-lowering effect of *Lactobacillus pentosus *KF923750 was examined on the lipid profile of rabbits. Total cholesterol, LDL-C, and TGs levels were significantly decreased, while no significant change was seen in HDL-C levels (100). *Lacticaseibacillus rhamnosus *BFE5264 could reduce the serum levels of cholesterol that were accompanied by changes in gut microbiota and production of SCFA (101). In Asan-Ozusaglam *et al*. (2019) study, the cholesterol reduction ability of *L. fermentum* strains isolated from human breast milk varied from 34.84 to 91.15%. The supernatants showed higher cholesterol reduction ability compared with pellets (102). In an interesting study, Wang *et al*. (2019) demonstrated that *L.casei* pWQH01 and *L. plantarum* AR113 with high BSH activity are able to significantly reduce the serum cholesterol levels in hypercholesterolemic mice, whereas *L. casei* LC2W without BSH activity has a poor capability to reduce cholesterol (103).

***Bifidobacterium species***

*Bifidobacterium *species have shown cholesterol-lowering ability in various studies (104-109).* Bifidobacterium longum * (*B. longum*) and *B. bifidum* are the most common *Bifidobacterium* species that have been used to evaluate the cholesterol-lowering effects (48, 104-106, 109). In a study, consumption of yogurt containing *Bifidobacterium pseudocatenulatum* G4 or *B. longum* BB536 for 8 weeks led to improvements in the lipid profile and increased fecal excretion of BAs in rats compared with the control groups (106). Administration of *B. longum* BB536 resulted in a significant reduction of total cholesterol, lipid deposition of liver and adipocyte size, and positively affected the function of kidney and liver in hypercholesterolaemic Sprague-Dawley rats. Cholesterol-lowering effect of* B. longum* BB536 significantly increased in the presence of inulin (105). Also, *B. animalis* subsp. Lactis F1-7 (77) and *B. pseudolongum* (107) have reduced the serum cholesterol levels in mice. It has been shown that the cholesterol-lowering efficacy of *Bifidobacterium* species increases in combination with *Lactobacilli* species (108). Ranji *et al*. (2019) reported that oral consumption of *B. bifidum *and *Lactobacilli *leads to a significant decrease in the TGs and LDL-C in mice (*P<*0.005) (104). In a study, *B. longum* strains (CCFM 1077, I3, J3, and B3) showed different levels of cholesterol reduction which raised the strain-specific effects in reducing cholesterol (109). BSH activity is the most common mechanism investigated in *Bifidobacterium* species (44, 48). 

***Clinical studies on cholesterol-lowering***

Several clinical studies have been performed on the cholesterol-lowering effects of *Lactobacilli* and *Bifidobacterium *species which are summarized in Table 1. Clinical studies are different based on country, bacteria, sample size, duration, dosage, delivery, subjects, and outcome. 

Most clinical studies have been performed on hypercholesterolemic individuals. In two studies, Fuentes *et al*. evaluated the cholesterol-lowering effect of *L.* *plantarum *strains in hypercholesterolemic adults. They reported that administration of *L. plantarum*-containing capsules significantly improves the lipid profile of patients. They also declared that the biofunctionality of *L. plantarum* strains has better effects in patients with higher levels of cholesterol (110, 111). Another study showed that administration of *L. fermentum*-containing capsules has no significant efficacy on serum lipids in individuals with elevated serum cholesterol (112). In Lewis *et al*. (2005) study, although *L. acidophilus* significantly reduced *in vitro* cholesterol, no effect was seen in the serum lipid profile of hypercholesterolaemic volunteers (113).

Also, a powder containing* L. curvatus* HY7601 and *L. plantarum* KY1032 has reduced TGs levels in hypertriglyceridemic individuals. (114). In addition to capsules and powder, other delivery methods have been used to evaluate the cholesterol-lowering efficacy of *Lactobacilli *and *Bifidobacterium*, especially in hypercholesterolemic individuals. Yogurt and fermented milk which contain *Lactobacilli *or/and *Bifidobacterium* are the most common delivery methods. It has been shown that probiotic milk formula (PMF) containing *L. acidophilus *(La5), *L. casei *(TMC), and *B. lactis *(Bb12) can significantly reduce the total cholesterol and LDL-C of hypercholesterolemic volunteers. (115). Also, milk fermented by *B. longum* strain BL1 (116) and fermented milk containing *L. acidophilus* l1 (117) have reduced the serum concentrations of total cholesterol, LDL-C, and TGs, while not changing the level of HDL-C. Combination of soy with a probiotic containing *Lactobacillus acidophilus*, *B. bifidus*, and *Lactobacillus* GG resulted in a significant reduction of total cholesterol and LDL-C in mildly hypercholesterolaemic subjects (118). It has been found that in addition to *in vitro* and animal studies, BSH-active *Lactobacilli *and *Bifidobacterium* have remarkable effects on cholesterol in human studies. *L. plantarum* ECGC 13110402 (119) and* L. reuteri* NCIMB 30242 (120), strains with high BSH activity, have demonstrated significant improvement in the lipid profile of hypercholesterolaemic subjects.

After hypercholesterolemic subjects, most clinical studies have been done on the lipid profile of healthy subjects. It seems that *Lactobacilli *and *Bifidobacterium* are more effective in hypercholesterolemic subjects than in healthy subjects. Although yogurt containing *L. acidophilus* La5 and *B. lactis* Bb12 (121) and probiotic yogurt fermented with *L. acidophilus* and *B. lactis* (122) have decreased total cholesterol, they had no significant effects on TGs and LDL-C in healthy adults. Also, in other studies, *B. animalis* subsp. lactis BB-12 and *L. acidophilus* La5 did not significantly improve the lipid profile including total cholesterol, LDL-C, HDL-C, and TGs in healthy participants (123, 124).

In type 2 diabetic patients, consumption of probiotic yogurt containing *L. acidophilus *and *B. lactis *resulted in a 4.54% decrease in total cholesterol and a 7.45% in LDL-C, and a significant reduction in the total cholesterol:HDL-C ratio and LDL-C:HDL-C ratio (125), but consumption of probiotic-containing *lactobacilli* did not reduce the serum TGs concentration, total cholesterol, and LDL-C levels (126). 

It has been shown that* L. acidophilus *DDS-1, *B. longum,* and *L. plantarum* 299v do not change significantly the serum cholesterol levels in postmenopausal women (127) and smokers (128), respectively. 

Although improved lipid profiles are often associated with decreased serum levels of LDL-C and total cholesterol and increased HDL-C, in many clinical studies many cases were unchanged in HDL-C. Even in a study by Rerksuppaphol *et al*. (2015) (129), HDL-C decreased which seems illogical, and the author did not explain it.

***Effects on inflammation and oxidative stress and trimethylamine-N-oxide (TMAO)***

**Figure 4:**
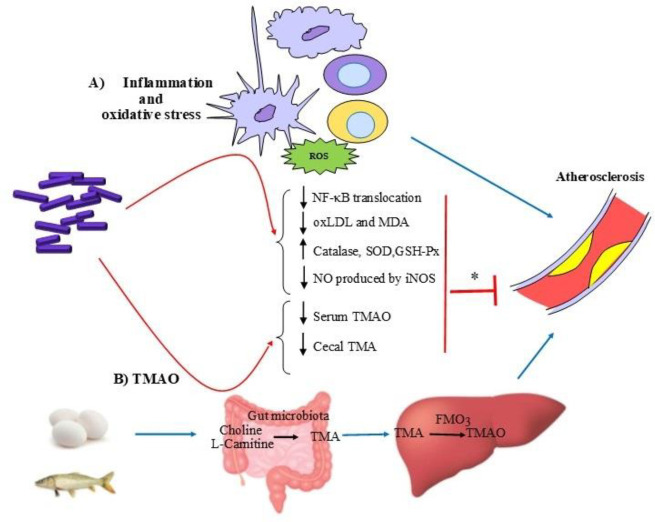
Anti-atherosclerotic effects of *Lactobacilli* and *Bifidobacterium* through improving A) inflammation and oxidative stress and B) TMAO. ROS, reactive oxygen species; NF-KB, nuclear factor kappa B ; ox-LDL, oxidized low-density lipoprotein ; MDA, Malondialdehyde; SOD, super oxide dismutase; GSH-Px, glutathione peroxidase ; NO, Nitric oxide ; iNOS, inducible nitric oxide synthase ; TMA, trimethylamine; TMAO, Trimethylamine N-oxide; FMO3, flavin-containing monooxygenases. *, inhibition of atherosclerosis

Hypercholesterolemia has been generally considered an important cause of atherosclerosis (130). However, other studies have demonstrated that despite the reduction of serum level of LDL-C more than 50% of cardiovascular risk still remains (131, 132). So, we have to look for other causes of this important disease, one of which is inflammation and oxidative stress and the other is trimethylamine N-oxide (TMAO). There are studies that have shown the beneficial effects of probiotics on these atherogenic factors (21, 22). Figure 4 shows the hypothesized anti-atherosclerotic mechanisms of *Lactobacilli *and *Bifidobacterium* on inflammation and oxidative stress and TMAO based on performed studies.

***Inflammatory and oxidative stress factors***

Studies have shown that in addition to hypercholesterolemia, inflammation and oxidative stress also play a vital role in the progression of atherosclerosis (21). Atherosclerosis is regulated by various immune cells such as macrophages, lymphocytes, and dendritic cells, and causes foam cells to form in the subendothelial region by the deposition of cholesterol. Atherosclerotic plaques express a complex network of proinflammatory cytokines, including interleukins (IL), tumor necrosis factor-alpha (TNF-α), and interferons (21, 133). IL-1β has substantial effects on cell types that cause atherosclerotic plaques (21). TNF-α produced by smooth muscle cells, macrophages, and endothelial cells of atherosclerotic arteries, has potent estrogenic effects via stimulating the formation of new vessels, adhesion of leukocytes to endothelial cells, chemotaxis, and developing atheroma features. In addition, inhibitors of nuclear factor kappa B (IκB) proteins including IκBα, β, and γ are regulatory proteins that bind to nuclear factor kappa B (NF-κB) and prevent transcription and nuclear translocation of NF-κB, a factor with an important role in the expression of proinflammatory cytokines. 

Oxidative stress is the overproduction of reactive oxygen species (ROS) in such a way that anti-oxidant systems are unable to control or repair their damage (134). ROS including free oxygen radicals, oxygen ions, and peroxides at moderate concentrations play critical roles in the regulation of various cell functions and biological processes such as vascular tone, oxygen sensing, cell growth and proliferation, apoptosis, and inflammatory responses (135). Uncontrolled production of ROS resulted in oxidative stress and endothelial dysfunction. Oxidative stress plays an important role in the atherosclerosis process (135). 

Malondialdehyde (MDA) and oxidized low-density lipoprotein (oxLDL) are oxidative biomarkers that arise following oxidative stress and lipid peroxidation by ROS (134, 136). It has been shown that ox-LDL plays a key role in several steps of atherogenesis and also inhibits endothelial nitric oxide synthase (eNOS) activity in endothelial cells (134, 137). In several studies, ox-LDL was elevated in the plasma of atherosclerotic patients (134, 138). Superoxide dismutase (SOD), catalase, glutathione peroxidase (GSH-Px), paraoxonase (PON), and thioredoxins are major anti-oxidants in the vascular wall (4, 134).

Nitric oxide (NO) synthesized by endothelial nitric oxide synthase (eNOS) and/or neuronal nitric oxide synthase (nNOS) have anti-atherosclerotic activity, whereas NO produced by inducible nitric oxide synthase (iNOS) plays a pro-atherogenic role (135). Activation of iNOS can form uncoupled eNOS which is a ROS generator and contributes to atherogenesis (134). Inflammation, oxidative stress, bacterial endotoxins, and sepsis, activate iNOS, which produces Ca^2+^-independent NO at a rate 1,000-fold greater than that of eNOS (137, 139).

Various studies have demonstrated the ability of *Lactobacilli* and *Bifidobacterium* species in modulating inflammation and oxidative stress (140-143). In a study, Hassan *et al*. (2020) reported that *L. plantarum* ATCC 14917 is able to exert its anti-atherosclerotic effects via modulation of proinflammatory cytokines and oxidative stress. They observed that feeding *L. plantarum* ATCC 14917 to Apo E−/− mice daily for 12 weeks had no effect on body weight and lipid profile, but significantly inhibited atherosclerotic lesion formation. In addition, the oxidative stress factors such as oxLDL and MDA, as well as inflammatory factors including NF-κB and IL-1β levels were significantly reduced, whereas the level of SOD was induced. Furthermore, administration of *L. plantarum* ATCC 14917 significantly attenuated the IκBα protein degradation and inhibited translocation of the p65 subunit of NF-κB, and also modulated the gut microbiota composition in ApoE−/− mice (21). In another study, Fang *et al*. (2019) evaluated the effect of *L. rhamnosus* GR-1 on atherosclerotic plaque formation in ApoE-/- mice fed with a high-fat diet. Administration of GR-1 had no efficacy in body weight or serum levels of lipid, but reduced the progression of atherosclerosis and plaque formation. Also, administration of GR-1 reduced the values of oxLDL and MDA, inhibited the translocation of NF-κB p65, and diminished the expression of inflammation cytokines TNF-α and IL-6 (132). Also, Chen *et al*. (2013) showed similar results when using *L. acidophilus* ATCC 4356 (142). Treatment with* L. fermentum* MTCC: 5898 had beneficial effects on inflammation and oxidative stress in hypercholesterolemic Wistar rats. It significantly reduced atherogenic index, coronary artery risk index, lipid peroxidation, and mRNA expression of inflammatory cytokines (TNF-α and IL-6) and significantly increased anti-oxidative enzymes (catalase, SOD, and GSH-Px) (144). In another study, the anti-oxidative effects of *L. casei *Zhang on hyperlipidemic rats were evaluated. Treatment with* L. casei *Zhang significantly decreased MDA levels and increased SOD and GSH-Px in the serum and liver of rats. Authors also declared that the anti-oxidative effect of *L. casei *Zhang is more effective in hyperlipidemic rats compared with normal rats, and application of different therapeutics doses have variant effects on hyperlipidemic rats (145). Kim *et al*. (2013) examined the inhibitory effects of (pLTA)- lipoteichoic acid (LTA) of *L. plantarum*- on atherosclerotic inflammation. They observed that pLTA prevented the production of proinflammatory cytokines and NO in lipopolysaccharide (LPS)-stimulated cells. The authors declared that the inhibitory effect of pLTA is due to inhibition of NF-κB and activation of mitogen-activated protein kinases (MAP kinases). They concluded that their results emphasize the role of pLTA in suppressing atherosclerotic inflammation (146).

***Trimethylamine-N-oxide (TMAO)***

It has been shown that one of the causes of atherosclerosis and cardiovascular diseases is linked to the high level of TMAO (147). TMAO is oxidized from trimethylamine (TMA) by hepatic flavin-containing monooxygenase3 (FMO3). TMA is produced from dietary L-carnitine, phosphatidylcholine, choline, and betaine by intestinal microbiota (22). Precursors of TMA are highly present in foods of animal origin such as red meat, dairy products, and eggs. Seafood such as fish has more carnitine and choline and also TMA and TMAO compared with other foods (148). The serum level of TMAO is dictated by several factors such as gut microbiota, diet, drug administration, and liver FMO3 activity (149). TMAO mediated by intestinal microbiota has been a promising target for the treatment of atherosclerosis (150).

Although the atherosclerotic mechanisms of TMAO are not fully understood, some hypotheses are related to cholesterol metabolism. TMAO can activate SHP and FXR and down-regulate enzymes that synthesize BAs including CYP7A1 and CYP27A1 (151). Activation of FXR can decrease reverse cholesterol transport and induce FMO3 which can lead to atherosclerosis. It has been shown that TMAO is able to reduce the expression of NPC1L1 and ABCG5/8 (152) and also up-regulate ABCG5/8 in the small intestine (153). Some studies have shown the down-regulation effect of TMAO on ABCA1 and ABCG1 (152-154) and scavenger receptor A (SRA) and cluster of differentiation 36 (CD36) (155) expression in macrophages which cause the formation of foam cells and atherosclerotic plaques. It has been suggested that supplementation with probiotic strains such as *Lactobacilli* and *Bifidobacterium* strains might change TMAO levels. Qiu *et al*. (2018) showed that *L. plantarum *ZDY04 significantly reduced serum TMAO and cecal TMA levels by modulating the gut microbiota in apolipoprotein-E knockout (ApoE−/−) mice (a mouse model of atherosclerosis). They also reported that the reduction of serum TMAO and cecal TMA was not due to influencing the expression levels of hepatic FMO3 and metabolizing choline, TMA, and TMAO. In this study, *L. plantarum *ZDY04 could significantly inhibit the development of TMAO-induced atherosclerosis in ApoE−/− mice as compared with the control (22).

**Table 1:** Clinical studies of cholesterol-lowering efficacy of *Lactobacilli* and *Bifidobacterium* species

**Study **	**Country **	**Bacteria **	**Sample size**	**Duration** **(weeks)**	**Dosage **	**Delivery **	**Subjects **	**Outcome **	**Ref.**
Ejtahed *et al*. (2011)	Iran	*L. acidophilus* La5 and *B. lactis *Bb12	60	6	300 g daily	Yogurt	Type 2 diabetic patients	Significant decrease in total cholesterol and LDL-C, no significant changes in TG and HDL-C	(125)
Mazloom *et al*. (2013)	Iran	*L. acidophilus, L. bulgaricus,* *L. bifidum, and L. casei*	34	6	1500 mg probiotic capsules twice daily	Capsule	Type 2 diabetic patients	No significant difference in serum levels of TG, total cholesterol, LDL-C, and HDL-C levels	(126)
Fuentes *et al*. (2016)	Spain	*L. plantarum*	60	12	1.28–3.01×10^9 ^CFU daily	Capsule	Hypercholesterolemic subjects	Significant reduction in LDL-C, total-C, LDL-C:HDL-C ratio, oxidized LDL and triglycerides, and a significant increase in HDL-C	(110)
Xiao *et al*. (2003)	Japan	*B. longum* strain BL1	32	4	>10^8^ CFU/ml, 300 ml daily	Yogurt	Healthy subjects	Significant decrease in serum total cholesterol	(116)
Lee *et al*. (2017)	USA	*B. animalis *subsp. lactis BB-12	30	4	Each smoothie/capsule contained 3.16 × 10^9^ CFUs/ day	Yogurt/capsule	Healthy subjects	No significant change in total cholesterol, LDL-C, HDL-C, and TGs	(123)
Chiu *et al*. (2021)	Taiwan	*L. acidophilus (La5), L. casei (TMC), B. lactis *(Bb12)	40	10	2 ×10^6^ CFU/g of each strain	Powder	Healthy mild hypercholesterolemic subjects	Significantly reduced (*P*<0.05) the levels of total cholesterol and LDL-C	(115)
Greany *et al*. (2004)	USA	*L.acidophilus DDS-1* *and B. longum)*	37	6	10^9^ CFU	Capsule	Mildly hypercholesterolemic postmenopausal women	Significant reduction in serum TC, LDL-C, and TG, and increase in HDL-C	(127)
Ahn *et al*. (2015)	Republic of Korea	*L. curvatus HY7601 and L. plantarum KY1032*	128	12	2 g/day of a powdered supplement containing *L. curvatus *HY7601 and *L. plantarum* KY1032	Powder	Hypercholesterolemic subjects	Significant reduction in TGs and increases in the plasma apo A-V	(114)
Sadrzadeh-Yeganeh *et al*. (2009)	Iran	*L. acidophilus* La5* and B. lactis *Bb12	90	6	Daily 300 g probiotic yogurt containing *Lactobacilli *species.	Yogurt	Healthy subjects	No difference in TG and LDL-cholesterol, but a decrease in total cholesterol:HDL-cholesterol ratio, and an increase in HDL-cholesterol	(121)
Simons *et al*. (2006)	Australia	*L. fermentum*	44	10	2×10^9^ CFU/capsule, 2 capsules twice daily	Capsule	Hypercholesterolemic subjects	No significant changes in total cholesterol, HDL cholesterol, or TGs	(112)
Rerksuppaphol *et al*. (2015)	Thailand	*L. acidophilus*,* B. bifidum*	64	6	10^9^ CFU/capsule, thrice daily	Capsule	Hypercholesterolemic subjects	TC levels in the probiotics group decreased	(129)
Naruszewicz *et al*. (2002)	Sweden	*L. plantarum* 299v	36	6	5×10^7^ CFU/ml, 400 ml daily	A rose-hip drink	Smoker	no significant changes in total cholesterol, triacylglycerol, and lipoprotein	(128)
Lewis SJ (2005)	UK	*L. acidophilus*	79	6	3 × 10^10^ CFU, thrice daily	Capsule	Hypercholesterolemic subjects	there were no changes in serum lipids seen throughout the study	(113)
Larkin *et al*. (2009)	Australia	*L. acidophilus*; *B. bifidus* and L. GG	15	5	3×10^8^ CFU daily	Yogurt	Hypercholesterolemic subjects	significantly decreased total cholesterol	(118)
Jones *et al*. (2012)	Canada	*L. reuteri* NCIMB 30242	127	9	2×10^9^ CFU/capsule, twice daily	Capsule	Hypercholesterolemic subjects	significantly reduced LDL-C and total cholesterol. HDL-C was unchanged	(120)
Ivey *et al*. (2015)	Australia	*L. acidophilus* La5; *B. animalis *subsp Lactis Bb12	a:77 b:79	6	3×10^9^ CFU daily	a: yogurt b: capsule	Overweight men and women	no significant change in serum total cholesterol LDLC, HDLC, or triglycerides	(124)
Fuentes *et al*. (2013)	Spain	*L. plantarum* CECT 7527, 7528 and 7529	60	a:6; b:12	1.2 × 10^9^ CFU daily	Capsule	Hypercholesterolemic subjects	Significant reduction in plasma total cholesterol, LDL-C, and oxidized LDL-C	(111)
Costabile *et al*. (2017)	UK	*L. plantarum* ECGC 13110402	46	12	2×10^9^ CFU/capsule, twice daily	Capsule	Hypercholesterolemic subjects	Significant reduction in LDL-C, TC, TG, and an increase in HDL-C	(119)
Ataie-Jafari *et al*. (2009)	Iran	*L. acidophilus*; *B. lactis*	14	6	2 ×10^6^ CFU/g, 300 g daily	Yogurt	Hypercholesterolemic subjects	Significant decrease in serum total cholesterol	(122)
Anderson *et al*. (1999)	USA	*L. acidophilus* L1	40	4	10^7^ CFU/g, 200 g daily	Yogurt	Hypercholesterolemic subjects	Significant reduction of serum cholesterol concentration	(117)

## Discussion

Here we reviewed the effects of *Lactobacilli* and *Bifidobacterium *on important risk factors of atherosclerosis including hypercholesterolemia, inflammation, oxidative stress, and TMAO. Given that hypercholesterolemia is one of the most important risk factors for atherosclerosis, a significant part of the present review is devoted to the cholesterol-lowering effects of these probiotics *in vitro*, animal, and human studies. This review showed that *in vitro* and animal studies have shown a greater cholesterol-lowering effect than human studies. The reasons for these differences may include differences in bacterial species and strains, duration of the studies, dosage, delivery method, studied individuals, and also the used composition. The present review mentioned various possible cholesterol-lowering mechanisms of *Lactobacilli* and *Bifidobacterium, *of which, deconjugation of bile by the BSH enzyme is more studied and well-known. In reviewed studies, *Lactobacilli* and *Bifidobacterium* strains that have shown high BSH activity significantly reduced the cholesterol levels. This mechanism is not yet clear enough, so further studies are needed to determine the exact effects of the BSH enzyme. In reviewed clinical studies, the cholesterol-lowering ability of probiotic strains in hypercholesterolemic individuals was more promising than in other individuals. It can be found out that although several clinical studies have been performed on cholesterol reduction by *Lactobacilli* and *Bifidobacterium *strains, most of them have only examined their effectiveness, and unlike *in vitro* and animal studies, the mechanism of cholesterol reduction has not been studied. Therefore, more studies are needed in both *in vitro* and *in vivo* (animal and human) conditions to shed more light on the effects and mechanisms of cholesterol-lowering. However, other studies have shown that despite reduced serum LDL-C levels, more than 50% of cardiovascular risks persist which raises the involvement of other factors in causing this disease. Indeed, inflammation and oxidative stress are now widely considered to play a vital role in atherosclerotic progression. Thus, in addition to the cholesterol-lowering effect, the effects of *Lactobacilli* and *Bifidobacterium* on inflammation and oxidative stress have attracted the attention of researchers and fortunately yielded promising results. An important point in our review is that although some *Lactobacilli* and *Bifidobacterium* strains may not show significant cholesterol-lowering effects in human studies, it should be hoped that these strains can have anti-atherosclerotic effects via reduction of inflammation, oxidative stress, and TMAO. 

## Conclusion

This review presented the effects of two well-known bacterial probiotics including *Lactobacilli* and *Bifidobacterium* on the most important risk factors for atherosclerosis such as hypercholesterolemia, inflammation, oxidative stress, and TMAO. Our review showed that these microorganisms are able to reduce cholesterol significantly *in vitro*, in animals and humans, although this ability is greater *in vitro* in animals than in humans. These useful microorganisms are also able to reduce the risk of atherosclerosis via reducing the serum levels of TMAO by improving the gut microbiota. Also, it can be concluded that these probiotics can diminish the risk of atherosclerosis by inhibiting inflammation and oxidative stress, an interesting and promising mechanism. Finally, we propose further studies to elucidate the effects and mechanisms of cholesterol-lowering, serum TMAO reduction, and inhibition of inflammation and oxidative stress by *Lactobacilli* and *Bifidobacterium*.

## Authors’ Contributions

MA searched the databases and wrote the manuscript; HEGG edited the manuscript; RR designed and supervised the study.

## Conflicts of Interest

There are no conflicts of interest.
